# Uncover spatially informed variations for single-cell spatial transcriptomics with STew

**DOI:** 10.1093/bioadv/vbae064

**Published:** 2024-05-29

**Authors:** Nanxi Guo, Juan Vargas, Samantha Reynoso, Douglas Fritz, Revanth Krishna, Chuangqi Wang, Fan Zhang

**Affiliations:** Biostatistics and Informatics PhD Program, University of Colorado Anschutz Medical Campus, Aurora, CO 80045, United States; Department of Biomedical Informatics, Center for Health Artificial Intelligence, University of Colorado Anschutz Medical Campus, Aurora, CO 80045, United States; Department of Biomedical Informatics, Center for Health Artificial Intelligence, University of Colorado Anschutz Medical Campus, Aurora, CO 80045, United States; MPH Biostatistics, University of Colorado Anschutz Medical Campus, Aurora, CO 80045, United States; Department of Biomedical Informatics, Center for Health Artificial Intelligence, University of Colorado Anschutz Medical Campus, Aurora, CO 80045, United States; Computational Bioscience PhD Program, University of Colorado School of Medicine, Aurora, CO 80045, United States; Department of Biomedical Informatics, Center for Health Artificial Intelligence, University of Colorado Anschutz Medical Campus, Aurora, CO 80045, United States; Medical Scientist Training Program, University of Colorado Anschutz Medical Campus, Aurora, CO 80045, United States; Department of Biomedical Informatics, Center for Health Artificial Intelligence, University of Colorado Anschutz Medical Campus, Aurora, CO 80045, United States; Division of Rheumatology, Department of Medicine, University of Colorado Anschutz Medical Campus, Aurora, CO 80045, United States; Department of Immunology and Microbiology, University of Colorado Anschutz Medical Campus, Aurora, CO 80045, United States; Department of Biomedical Informatics, Center for Health Artificial Intelligence, University of Colorado Anschutz Medical Campus, Aurora, CO 80045, United States; Division of Rheumatology, Department of Medicine, University of Colorado Anschutz Medical Campus, Aurora, CO 80045, United States

## Abstract

**Motivation:**

The recent spatial transcriptomics (ST) technologies have enabled characterization of gene expression patterns and spatial information, advancing our understanding of cell lineages within diseased tissues. Several analytical approaches have been proposed for ST data, but effectively utilizing spatial information to unveil the shared variation with gene expression remains a challenge.

**Results:**

We introduce STew, a Spatial Transcriptomic multi-viEW representation learning method, to jointly analyze spatial information and gene expression in a scalable manner, followed by a data-driven statistical framework to measure the goodness of model fit. Through benchmarking using human dorsolateral prefrontal cortex and mouse main olfactory bulb data with true manual annotations, STew achieved superior performance in both clustering accuracy and continuity of identified spatial domains compared with other methods. STew is also robust to generate consistent results insensitive to model parameters, including sparsity constraints. We next applied STew to various ST data acquired from 10× Visium, Slide-seqV2, and 10× Xenium, encompassing single-cell and multi-cellular resolution ST technologies, which revealed spatially informed cell type clusters and biologically meaningful axes. In particular, we identified a proinflammatory fibroblast spatial niche using ST data from psoriatic skins. Moreover, STew scales almost linearly with the number of spatial locations, guaranteeing its applicability to datasets with thousands of spatial locations to capture disease-relevant niches in complex tissues.

**Availability and implementation:**

Source code and the R software tool STew are available from github.com/fanzhanglab/STew.

## 1 Introduction

Single-cell transcriptomics are revolutionizing our comprehension of transcriptional heterogeneity by providing high-resolution view of transcriptome at the single-cell level for complex diseases, such as tumors, autoimmune disorders, neurology diseases (Shalek and Benson 2017, Stubbington *et al.* 2017, Papalexi and Satija 2018). However, single-cell transcriptomics typically requires dissociation of tissues into single cells, losing the spatial context of where each cell resides within the original tissue. Pinpointing the exact locations where the key cell phenotypes drive tissue and disease pathogenesis remains largely unclear. The recent emergence of spatial transcriptomics (ST) technologies has bridged this gap, facilitating concurrent measurements of gene expression with spatial localization, preserving the spatial information through the analysis of the intact tissue samples (Larsson *et al.* 2021, Moses and Pachter 2022). This spatial awareness provides a deeper understanding of the intricate relationships between the cells and their neighbors, unlocking new insights into the functions and mechanisms of cellular phenotypes, and thereby catalyzing the progression of innovative therapeutic strategy development.

Multiple cutting-edge ST technologies have been developed, varying significantly in aspects such as resolution and throughput (Larsson *et al.* 2021, Moses and Pachter 2022, Williams *et al.* 2022, Vandereyken *et al.* 2023). For example, the 10× Genomics Visium has the capability to analyze tens of thousands of genes on thousands of locations by spatial barcoding at 55-µm resolution (Ståhl *et al.* 2016). In parallel, Slide-seq (Rodriques *et al.* 2019) employs arrays comprised 10-μm barcoded beads followed by an improved version Slide-seq V2 (Stickels *et al.* 2020). *In situ* sequencing enables the measurement of the entire transcriptome at a single-cell resolution (Ke *et al.* 2013), such as 10× Xenium (Janesick *et al.* 2023). Moreover, MERFISH, a technology grounded in single-molecule fluorescent *in situ* hybridization (smFISH), demonstrates the capacity to detect hundreds to tens of thousands of genes at subcellular precision (Chen *et al.* 2015, Moffitt *et al.* 2016). While these ST technologies capture the abundant molecular information at the cellular or even subcellular resolutions, it introduces computational challenges in the sensitivity and robustness of down-stream data analysis. Typically, ST data is processed using exploratory data analysis techniques derived from single-cell transcriptomics, utilizing tools such as Seurat that have been enhanced with functionalities to facilitate the visualization of gene expression in spatial contexts. The phenotypes of many immune and mesenchymal cells are influenced by their locations within a tissue context. Thus, explicitly incorporating spatial information into the computational analysis necessitates the integration of spatial coordinates with gene expression variation through advanced joint modeling, which is essential for a more comprehensive dissection of biological tissue diversity.

While several methods have been developed to use both gene expression and spatial coordinates to analyze ST data, it remains a computational challenge given the complexity of disease tissue and deriving biologically meaningful phenotypes. For example, stLearn (Pham *et al.* 2023) introduces a normalization method that incorporates spatial location and tissue morphology to adjust gene expression values. However, stLearn requires the availability of H&E image; in their absence, it resorts to employing single model-based dimensionality reduction methods such as principal component analysis (PCA) and Louvain clustering. MERINGUE constructs a weighted graph that combines spatial and transcriptional similarities (Miller *et al.* 2021). This approach exploits spatial cross-correlation with gene expression patterns, restricting inferred interactions to short-range regions only. Furthermore, SpaceFlow (Ren *et al.* 2022) amalgamates the pseudotime concept with the spatial locations of cells using spatially regularized deep graph networks to elucidate cellular spatiotemporal patterns. Other platforms, such as Giotto (Dries *et al.* 2021), provide analytical frameworks with end-to-end toolboxes that utilize spatial information. However, these approaches often face trade-offs between their capability in capturing shared biological variations and computational scalability.

In this study, we delve into the advantages of leveraging spatial information to unveil the shared variation with gene expression space through the lens of representation learning (Li *et al.* 2019). Our hypothesis is based on the idea that neighboring cells are more likely to share similar transcriptional identities. Here, we present STew, a Spatial Transcriptomic multi-viEW representation learning method grounded in sparse canonical correlation analysis and graph representation, to jointly analyze spatial information and gene expression in a scalable fashion. Within STew, we have incorporated a data-driven statistical framework to assess the goodness of fit for biological axes-specific genes. STew outputs distinct spatially informed cell gradients, robust spatially informed clusters, and a statistical goodness of model fit to uncover significant genes that reflect subtle spatial changes. To validate its efficacy, we benchmarked STew by applying it to six ST datasets, encompassing a range of tissues with manual annotations as ground truth and heterogeneous diseased tissue analyzed by single-cell and multi-cellular resolution ST technologies. We demonstrate that STew achieves superior accuracy in identifying spatial domains while ensuring spatial continuity and smoothness. Moreover, STew scales almost linearly with the number of spatial locations, guaranteeing its applicability to datasets with thousands of spatial locations to capture disease tissue heterogeneity. We have implemented STew as an open-source R software, accessible at github.com/fanzhanglab/STew.

## 2 Methods

### 2.1 Dataset

We analyzed six ST datasets, which are available from their original publications and described in detail as follows:

For the 10× Visium human dorsolateral prefrontal cortex (DLPFC) data, raw count matrix, histology image, and spatial data are accessible in the “spatialLIBD package” (https://research.libd.org/spatialLIBD/) (Maynard *et al.* 2021, Pardo *et al.* 2022). Manual annotation information for this dataset is also provided. The DLPFC data (Sample ID 151673), measured 33 538 genes on 3639 cell spots, has been served as a gold standard to test the performance of computational methods for ST data analyses.

We also analyzed the 10× Visium Mouse Brain Sagittal Anterior dataset which was obtained from https://satijalab.org/seurat/articles/spatial_vignette.html. This dataset comprises 31 053 genes and 2696 cell spots. For the Slide-seqV2 mouse hippocampus dataset, measured 4017 genes and 41 786 cell spots, which was accessible from the Broad Institute database “Single Cell Portal” (https://singlecell.broadinstitute.org/single_cell/study/SCP815/highly-sensitive-spatial-transcriptomics-at-near-cellular-resolution-with-slide-seqv2#study-download) (Stickels *et al.* 2020).

For the psoriasis skin ST dataset, it was acquired from lesional psoriasis skin affected by inflammatory disorder (Sample ID PP1). This dataset detects 33 539 genes and 1352 cell spots. This dataset is accessible at GEO GSE173706 and GSE225475 (Ma *et al.* 2023).

For the mouse main olfactory bulb (MOB) dataset, it was generated from a healthy mouse MOB using 10× Visium with well-established cell annotations, containing 260 genes and 14 828 cells. This data is accessible in the “STdeconvolve” package (https://jef.works/STdeconvolve/) as well.

The Mouse Brain Coronal single-cell resolution ST data was generated from 10× Xenium, a single-cell resolution platform. To evaluate the computational burden in analyzing the full coronal section of this dataset, we downsampled the cells from 130 870 cells based on different ratios to test the scalability of different numbers of cells involved. 247 genes were detected in this dataset. It is provided in the 10× Genomics link (https://www.10xgenomics.com/datasets/fresh-frozen-mouse-brain-for-xenium-explorer-demo-1-standard).

### 2.2 Overview of STew

**Figure 1.**
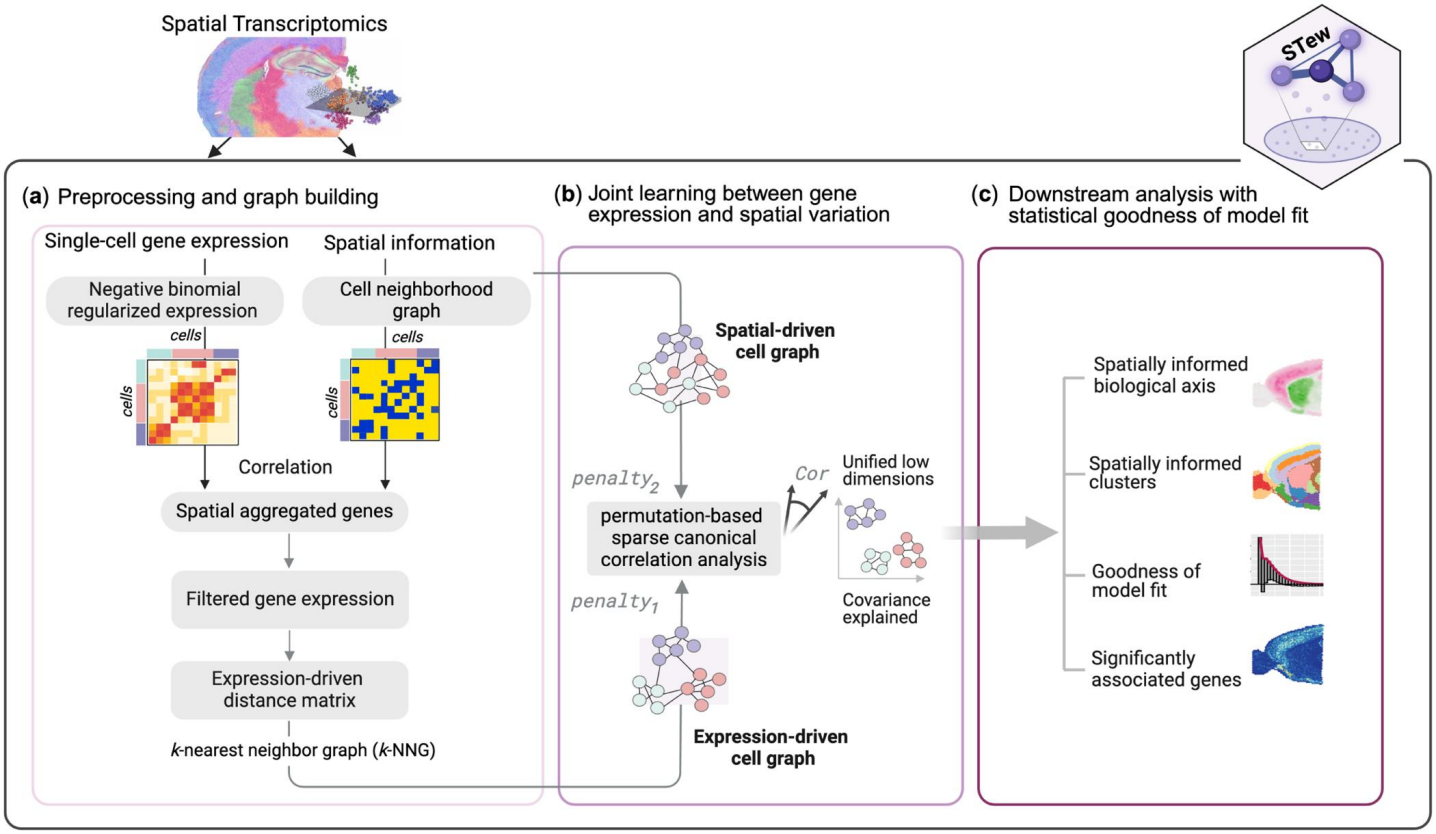
Overview of STew methodology. (a) Preprocessing and graph building. (b) Joint learning between gene expression and spatial variation. (c) Downstream analysis with statistical goodness of model fit.

We describe the methodology of STew as follows (Fig. 1). The main idea of STew is taking advantage of both spatial information and gene expression data by extracting shared information through multi-view representation learning. STew incorporates the graph-embedded information into the sparse canonical correlation analysis framework by explicitly modeling the sparsity to generate joint embeddings with maximal covariance.

We consider a ST data as $(Y,\;Z)\in \;R^{n\times p}\times R^{n\times 2}$ which denotes two views of collected data points on the same set of n observations, where p represents the number of features. $Y\in R^{n\times p}$ represents a gene expression matrix with n cell spots that measure p genes, and $Z\in R^{n\times 2}$ represent the same n spots that are distributed on the 2D physical coordinates.

### 2.3 Preprocessing and normalization on gene expression data

For the gene expression data Y, STew utilizes a regularized negative binomial regression model to normalize gene expression data to control for variation that may arise due to differences in sequencing depth or other potential technical inconsistencies (Fig. 1a).

### 2.4 Incorporating spatial data to build cell adjacency graph

For the spatial positional coordinates of the spots, STew characterizes cell–cell adjacency relationships by assigning binarized weight to each pair of cells using Delaunay triangulation, resulting in a binary adjacency matrix $M{\in \;R}^{n\times n}$ as a spatial-driven cell graph, where:

(1) Mij=1  if celli and cellj are adjacent0  if celli and cellj are not adjacent

### 2.5 Detection of spatially aggregated genes

STew defines spatially auto-correlated genes when their expressions vary across different regions of a tissue, often clustering in functionally related groups specific to that tissue region (Fig. 1a). STew detects such spatially aggregated genes by computing Moran’s *I* (Miller *et al.* 2021):

(2) $$I=\frac{N}{\sum_{i}^{n}\sum_{j}^{n}M_{ij}}\frac{\sum_{i}^{n}\sum_{j}^{n}M_{ij}(y_{i}-\bar{y})(y_{j}-\bar{y})}{\sum_{i}^{n}{\left(y_{i}-\bar{y}\right)}^{2}},$$

where $y_{i}$ denotes a normalized gene expression vector of gene i in Y. The adjacency matrix M describes positive spatial autocorrelations across n cells. The Benjamini–Hochberg procedure is used to correct for multiple testing and control for false discovery rate (FDR).

While global measure of spatial association (Moran’s *I*) provides a single value summarizing the overall spatial pattern of the gene expression, STew next filters out spatial patterns driven by small numbers of cells by Local Indicator of Spatial Association (LISA) (Anselin 2010). For each neighborhood of cell spot i:

(3) $$I_{i}=N\frac{\left(y_{i}-\bar{y})\sum_{j}^{n}M_{ij}(y_{j}-\bar{y}\right)}{\sum_{i}^{n}{\left(y_{i}-\bar{y}\right)}^{2}},$$

which is related to Moran’s *I* by:

(4) $$I=\sum_{i}^{n}\frac{I_{i}}{n}.$$

Specifically, STew further determines the spatial pattern scale based on the percentage of cells with a statistically significant LISA value. As a default setting, STew focuses on spatially heterogeneous genes with a *P*-value below the threshold of .05 that are driven by at least 5% of the cells. After such steps, we obtain an updated gene expression matrix, denoted as $Y\in R^{n\times q}$, where q represents the number of spatially aggregated genes.

### 2.6 Utilizing spatially aggregated genes to build cell graph

Next, STew characterizes cell–cell similarity matrix for $Y_{\mathrm{nxq}}$ by building a weighted K-nearest neighbors graph, where each node represents a cell, and an edge is drawn between a node and its k-nearest neighbors based on the Euclidean distance in terms of the gene expression (Fig. 1a). User may adjust the value of k to define the number of neighbors each cell is connected to, 200 by default.

The Euclidean distance is given by:

(5) $$d\left(c_{i},c_{j}\right)=\sqrt{{{(y}_{1i}-y_{1j})}^{2}+\cdots +{{(y}_{qi}-y_{qj})}^{2}},$$

where $c_{i}$ and $c_{j}$ denote a pair of cells, and $d\left(c_{i},c_{j}\right)$ is the Euclidean distance between cells in terms of q gene expression vectors.

The weighted KNN graph is given by:

(6) 0dc2,c1⋮dcn,c1 dc1,c20⋮⋯ ⋯⋮⋱⋯ dc1,cn⋮⋮0.

The weighted KNN graph is then converted into a binary adjacency matrix, denoted as $V\in R^{n\times n}$:

(7) Vij=1  if celli and cellj are connected 0  if celli and cellj are not connected,

where the connection is defined based on the statement that ${\mathrm{cell}}_{j}\;$is one of the k nearest neighbors of ${\mathrm{cell}}_{i}$.

In summary, this first step captures the relationships between cells in the high-dimensional space by converting the original gene expression into gene expression-driven adjacency matrix, denoted as $V\in R^{n\times n}$, and creating a spatial-driven cell neighborhood graph, denoted as $M{\in R}^{n\times n}$. These two adjacency matrices will be the inputs for the following step.

### 2.7 Permutation-based sparse canonical correlation analysis

Considering the different levels of sparsity and complexity of the two views, we incorporated penalized canonical coefficients by applying *L*_1_-norm penalties to the coefficients in Equation (8) (Fig. 1b). When dealing with high-dimensional problems, it has been approved that the within-set covariance matrices $\sum_{11}\;$and $\sum_{22}$ are usually replaced by identity matrix Ι to obtain decent results (Witten *et al.* 2009). Thus, we seek new pairs of sparse canonical coefficients $w_{1}\in \;R^{n\times 1}$ and $w_{2}\in \;R^{n\times 1}$ for M and V, respectively, by maximizing the correlation between $\left(Mw_{1},\;Vw_{2}\right)$ through this optimization problem:

(8) $$\begin{array}{c} \left(w_{1},w_{2}\right)=\mathrm{argmax}\;\mathrm{corr}\left(Mw_{1},\;Vw_{2}\right)\\ \begin{array}{c} =\mathrm{argmax}\frac{w_{1}^{T}\sum_{12}w_{2}}{\sqrt{w_{1}^{T}\sum_{11}w_{1}}\sqrt{w_{2}^{T}\sum_{22}w_{2}}}\\ \begin{array}{c} =\mathrm{argmax}\;w_{1}^{T}\sum_{12}w_{2}\\ \mathrm{subject}\;\mathrm{to}\;{\left||w_{1}|\right|}_{2}^{2}=1,\;{\left|\left|w_{2}\right|\right|}_{2}^{2}=1,\;|{\left|w_{1}|\right|}_{1}\;\leq \;c_{1},\;{\left||w_{2}|\right|}_{1}\;\leq \;c_{2}. \end{array} \end{array} \end{array}$$

Here, ${\left||\cdot |\right|}_{2}^{2}$ indicates the squared Frobenius norm (the sum of squared elements of the matrix), and ${\left||\cdot |\right|}_{1}=\sum_{i=1}^{n}\left|u_{i}\right|$ is known as Lasso. In ST data, we sometimes have a greater number of detected genes than that of spots, which means that these inverses of sample covariance matrices do not exist. Thus, we apply regularizations in the optimization.

In summary, this procedure yields sparse vectors $w_{1}$ and $w_{2}$ for $c_{1}\;$and $c_{2}$ chosen appropriately. Here, we restrict $c_{1}$ and $c_{2}$ to the range of $1\;\leq \;c_{1}\;\leq \;\sqrt{n}$ and $1\;\leq \;c_{2}\;\leq \;\sqrt{n}$, by scaling the $L_{1}$ regularization term with respect to M and V, resulting in penalty $0<\lambda_{1}\;\leq \;1$ and $0<\lambda_{2}\;\leq \;1$. Larger $L_{1}$ bound corresponds to less penalization.

The values of $c_{1}\;$and $c_{2}$ are determined through a parallelized permutation testing approach. For each candidate penalty parameter within the range of (0, 1), we randomly permute the rows of matrices M and V for k times to generate $M_{1}^{*},\;M_{2}^{*},\ldots ,\;M_{k}^{*}\;$and $V_{1}^{*},\;V_{2}^{*},\ldots ,\;V_{k}^{*}$. Then we run sparse CCA on each permuted dataset $(M_{i}^{*},\;V_{i}^{*})\;$to obtain factors ($w_{1i},\;w_{2i})$. We then compute the correlation $C_{i}=\mathrm{Corr}(M_{i}w_{1i},\;V_{i}w_{2i})$ and $C=\mathrm{Corr}(Mw_{1},\;Vw_{2})$, where (M, V) is from the original data and ${(w}_{1},\;w_{2})$ is the obtained factors from the original data. Lastly, we use Fisher’s transformation to convert the correlation to a random variable that is approximately normally distributed. Let Fisher(c) denotes the Fisher transformation of c, and then compute a z-statistic for Fisher(C) using:

(9) $$\frac{\mathrm{Fisher}\left(C\right)\;-\;\mathrm{mean}\left(\mathrm{Fisher}\left(C_{i}\right)\right)}{\mathrm{SD}\left(\mathrm{Fisher}\left(C\right)\right)\;+\;0.05}.$$

Here, 0.05 is added to the denominator to avoid division by zero issues. The magnitude of the z-statistic serves as an indicator of the significance of the corresponding tuning parameter value for matrices M and V. A larger absolute value of the z-statistic suggesting a superior fit of the sparse CCA model to the data, given that specific combination of tuning parameters. This implies a reliable and meaningful association between the matrices under consideration.

### 2.8 Downstream analysis with spatially informed canonical vectors

As outputs (Fig. 1c), we generate low-dimensional embeddings, termed as spatially informed canonical vectors (sCVs), that reflect the shared covariance. Furthermore, we detect cell-type clusters within a tissue via smart local moving algorithm by constructing a shared neighborhood graph based on sCVs. Other downstream analyses such as data visualization can be performed on these derived sCVs.

### 2.9 Statistical count modeling and goodness of model fit evaluation

As a downstream analysis, we can determine the transcripts that are significantly associated with sCVs or identified clusters. To tackle this problem, we should select the right statistical model for testing given the variety of means and SD of genes of interest. Thus, we incorporate statistical modeling comparison into STew by evaluating various count models to deal with the overdispersion attribute, including zero-inflated Poisson, negative binomial, zero-inflated negative binomial, Poisson generalized linear model (GLM), and Hurdle model, applying on raw counts instead of transformed values. We assess model performance using Akaike Information Criterion and Rootograms (Kleiber and Zeileis 2016) to determine the best-fitting model considering the issues of overestimate or underestimate. In addition, we evaluate the mean and variation captured under the gene expression space for each tested count model. Our framework also accounts for covariates in the likelihood ratio test by comparing the full model with the null model with the significance level of 0.05. We then generate coefficient and *P*-value for each tested gene, and report statistically significant genes as outputs.

### 2.10 Method benchmarking

We benchmarked STew with other state-of-the-art methods, including SpaceFlow V1.0.4, stLearn V0.4.12, and Seurat V5 with default parameters. We described the detailed parameters, inputs, and major analytical steps as follows.

Seurat PCA (Hao *et al.* 2023): Following the Seurat tutorial, we first normalized the raw gene expression count matrix using variance stabilizing transformation, which employs a regularized Negative Binomial regression model. Then, highly variable genes (HVGs) were obtained following the default parameter by the Seurat package. We then standardized the normalized expression value of each gene to have zero mean and unit SD. The standardized expression matrix was the input of PCA implemented in the Seurat function. For downstream analyses, including sensitivity evaluation, we extracted the top 12, 14, 16, 18, and 20 principal components following the tutorial in the Seurat package.

SpaceFlow (Ren *et al.* 2022): According to the SpaceFlow analytical pipeline, we input raw count data and spatial coordinates. Logarithmic transformation normalization was performed following the SpaceFlow tutorial. Subsequently, a selection of the top HVGs was made by default parameter. For the Deep GraphInfomax Model, a set of hyperparameters was utilized to optimize the training step. Spatial regularization strength was maintained at 0.1, while the latent dimension size varied through values 12, 14, 16, 18, and 20 to evaluate the sensitivity of the algorithm. Following the suggested optimal parameters, the learning rate was set at 1e-3, and the training regimen spanned up to 1000 epochs, with the max patience and minimum stop parameters established at 50 and 100, respectively.

StLearn (Pham *et al.* 2023): Following the pipeline of stLearn with default parameters, we first utilized SME (Spatial location, Morphology, and gene Expression) normalization strategy and performed unsupervised clustering, grouping similar spots into clusters and identifying subclustering options based on the spatial segregation within the tissue. PCA was used and relevant performance was evaluated by testing on different numbers of PCs, including top 12, 14, 16, 18, and 20 components. We further performed a nonlinear UMAP method for dimensionality reduction. Afterwards, a k-nearest neighbor graph is constructed with # clusters set based on the biological insights (e.g. seven clusters for the DLPFC dataset). On this graph’s adjacency matrix, we applied k-means neighbor clustering suggested by the stLearn pipeline.

### 2.11 Performance evaluation

To directly evaluate the information derived in the low-dimensional embeddings learned from different methods, we assess the performance of how the identified low-dimensional embeddings from STew, PCA, stLearn, and SpaceFlow could predict ground truth manual labels. For each combination of the top *n* sCVs (*n* = 4, 6, 8, 10, 12, and 14) derived from STew, we built a multinomial logistic regression model where sCVs as predictors and true manual labels are treated as outcome. Specifically, we trained the model on 70% of the cell spots and tested on the rest of 30% data. Alongside this model, a null model was also fit for each combination of components. The null model predicts the outcome without using any predictors and is solely based on the intercept, serving as a baseline to assess the improvement provided by including the low-dimensional components as predictors. Next, we calculated McFadden-adjusted pseudo-*R*^2^ to determine the model’s goodness of fit (McFadden 1972, Shang and Zhou 2022). To estimate the CIs, we further performed bootstrapping which involves repeatedly sampling with replacement from the training dataset, fitting models to these randomized samples, and recalculating the McFadden-adjusted pseudo-*R*^2^. The distribution of these recalculated samples is then used to estimate the CIs, providing a measure of uncertainty around the McFadden-adjusted pseudo-*R*^2^. This metric allowed us to evaluate the robustness of the model as it assesses the variability and stability of the model’s goodness of fit.

Moreover, we are able to evaluate the predictions on the test data regarding how the sCVs learned from the training data can be generalized to unseen data. A confusion matrix was constructed and compared to the ground truth labels. Thus, we calculated accuracy of the model and corresponding 95% CIs based on a binomial test. Sensitivity and specificity were calculated as well with estimated CIs by the same bootstrap method as previously noted.

We repeated this above evaluation analysis for the same numbers of top low-dimensional embeddings derived from PCA, stLearn, and SpaceFlow to compare the performance. We observed that STew increased its predictive power when using more sCVs, and outperformed the other tested methods. This analysis further indicates that the direct information learned from STew sCVs is spatial relevance and biologically meaningful regarding their ability to predict true spatial cluster labels.

To evaluate the performance of the clustering results, we measured the similarities of identified clusters for STew and other benchmarking methods with the true manual annotations using adjusted rand index (ARI):

(10) ARI=∑ijnij2-∑iai2∑jbj2/n212∑iai2+∑jbj2-∑iai2∑jbj2/n2,

where $a_{i}=\sum_{j}n_{ij}$, $a_{i}=\sum_{i}n_{ij}$.

Further, we quantified the continuity and smoothness of the identified spatial domains by calculating a percentage of abnormal spots (PAS) score. As the PAS score is calculated based on the proportion of spots with a cluster label that is different from at least six of its neighboring ten spots (Shang and Zhou 2022), lower PAS score indicates better spatial continuity and smoothness of the identified cluster.

## 3 Results

### 3.1 STew robustly recapitulates true manual annotations for human brain ST data

**Figure 2.**
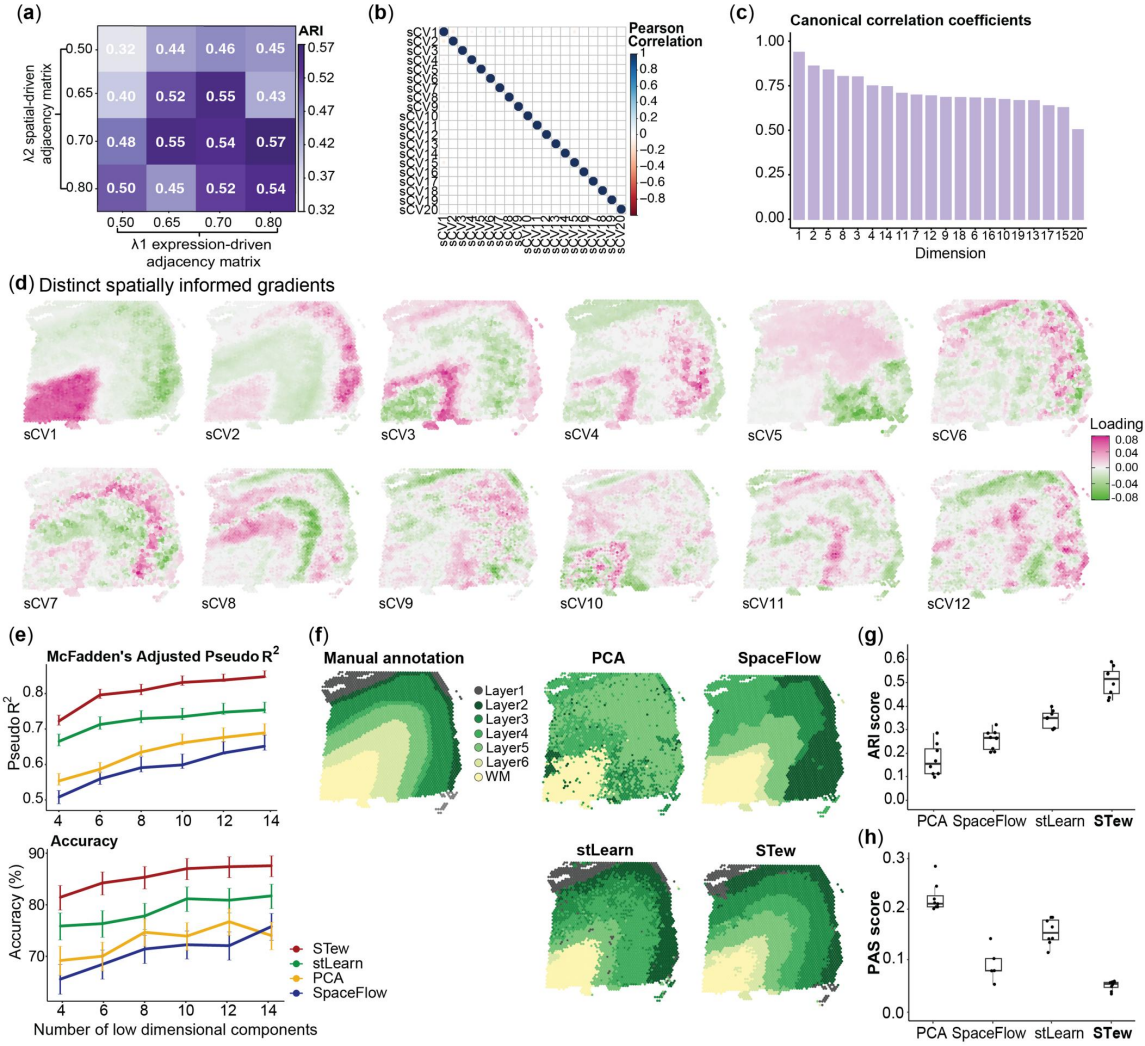
Benchmarking STew on 10× Visium dorsolateral prefrontal cortex data from DLPFC. (a) Adjusted rand index (ARI) scores to evaluate clustering accuracy by comparing with manual annotations. We tested different sparsity constraints in STew regarding their performance. (b) Pearson correlation coefficients of top 20 sCVs. (c) Barplot of canonical correlation coefficients of top 20 sCVs. (d) Distinct spatially informed gradients. (e) Evaluation of the low-dimensional components regarding their capability to predict the ground truth of tissue regions measured by McFadden-adjusted pseudo-*R*^2^ and accuracy. The 95% CIs are calculated based on bootstrapping. (f) Manual annotations of DLPFC in Slide 151673 and cell type clusters identified by PCA, SpaceFlow, stLearn, and STew using default parameters. (g) Clustering accuracy in recapitulating the true manual labels measured by ARI, with different numbers of low dimensions in each of the algorithms, in particular top 6, 8, 10, 12, 14, 16, 18, and 20 components. In the boxplot, the center line, box limits, and whiskers denote the median, upper, and lower quartiles, and 1.5 interquartile range, respectively. (h) Percentage of abnormal spots (PAS), the proportion of spots with a cluster label that is different from at list six of its neighboring ten spots.

We applied STew on human DLPFC data, measured 33 538 genes on 3639 cell spots, generated from 10× Visium (Maynard *et al.* 2021). We first identified sCVs and then performed graph-based clustering on them. In the STew framework, we automatically identified the optimal penalty parameters through cross-validation, measured the correlations between different sCVs, and identified the correlations that were reflected by each of the sCVs (Fig. 2a–d). Further, we estimated the optimal penalty parameters for sparse canonical correlation by comparing the clustering results with the true manual annotations using ARI (Fig. 2a), which suggests that the accuracies of clustering results are relatively robust but vary slightly according to the penalties (Supplementary Fig. S1). The variations among top 20 sCVs are orthogonal to each other (Fig. 2b), and each of the low-dimensional embeddings captures different shared variation between the gene expression and spatial regions (Fig. 2c). Consistent with these, we visualized each sCV at the spatial image, and different embeddings reflect distinct spatial region relevant niches (Fig. 2d).

We further benchmarked the performance of STew with other state-of-the-art methods. First, we evaluated the ability of the identified low-dimensional embeddings derived from each benchmarking method regarding the power to predict true manual labels. We built multinomial regression models and treated low-dimensional embeddings as predictors, then evaluated the performance by computing McFadden-adjusted pseudo-*R*^2^ and prediction accuracy (see Section 2). Bootstrapping was performed to estimate CIs. Our analysis revealed that sCVs derived from STew were highly predictive of the true spatial domains based on the original study (mean pseudo-*R*^2^=0.81, mean accuracy = 0.85, mean sensitivity = 0.81, and mean specificity = 0.97), outperformed those extracted from PCA, stLearn, and SpaceFlow (Fig. 2e, Supplementary Fig. S5b). Further, we compared STew-derived clusters, layer L1–L6 and white matter (WM), with the true manual annotations using ARI and found that STew (median ARI = 0.52) outperformed PCA (median ARI = 0.16), SpaceFlow (Ren *et al.* 2022) (median ARI = 0.27), and stLearn (median ARI = 0.35) regarding clustering accuracy (Fig. 2f and g). The ability to clearly recapitulate different spatially relevant niches is essential, given that many cell types or the expression of specific genes play pivotal roles in supporting or defining important biological processes within a tissue microenvironment. For example, layer 1 (L1), characterized by high expression levels of *FABP7* and *AQP4,* is recognized as a molecular layer enriched in synapses (Supplementary Fig. S7). Notably, *FABP7*, predominantly found in astrocytes, is known to regulate dendritic morphology and the formation of neuronal excitatory synapse (Ebrahimi *et al.* 2016). In contrast, WM is distinguished by a higher cell density of oligodendrocytes (Maynard *et al.* 2021). Neuronal cell subtypes, encompassing a variety of excitatory and inhibitory neurons, are typically associated with layers L2/L3, L4, and L5, showing a tendency for more layer-enriched expression among excitatory cells. Therefore, the identification of spatial niches allows for a more comprehensive understanding of how cell type-specific programs are linked to functional activities in the tissue context.

In addition, we measured the continuity and smoothness of the identified spatial domains by PAS score, which reveals that STew (median PAS = 0.06) is smoother and more continuous than PCA (median PAS = 0.21), stLearn (median PAS = 0.16), and SpaceFlow (median PAS = 0.08) (Fig. 2h). Lower PAS score here indicates a spot homogeneity within the identified clusters. After testing the effects of using different numbers of low-dimensional embeddings as inputs, we note that all the involved methods achieved consistent performances reflected by the ARI (Fig. 2e and g); STew achieves slightly better consistency than other methods regarding the smoothness of the identified spatial domains (Fig. 2g).

We have benchmarked STew on another ST dataset, mouse MOB generated from 10× Visium (Ståhl *et al.* 2016), where well-established manual labels are available. We further demonstrated that STew consistently achieved reliable results on heterogeneous tissue, and more robustly identified spatial regions (median ARI = 0.60) with spatial continuity (median PAS = 0.37) compared to PCA (median ARI = 0.28, median PAS = 0.47) and SpaceFlow (median ARI = 0.30, median PAS = 0.5), across a variety of low-dimensional embeddings (Supplementary Fig. S6).

### 3.2 STew automatically determines the best model of fit for significant gene identification on mouse brain datasets

**Figure 3.**
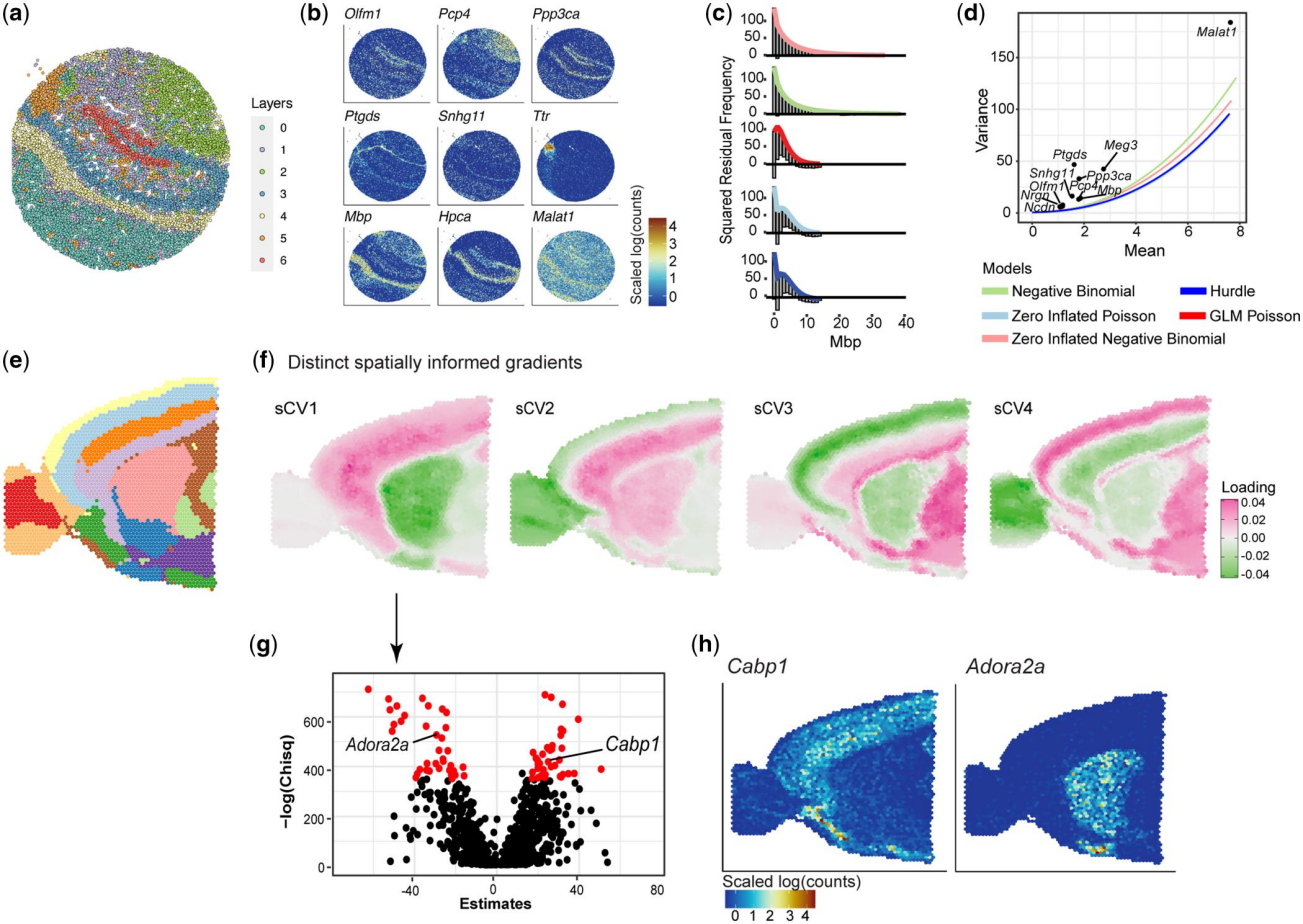
Applying STew on Slide-seqV2 mouse hippocampus and 10× Visium anterior section. (a) Identified cell type clusters of mouse hippocampus region on data from Slide-seqV2. (b) Gene expression distributions in the spatial domains that reflected spatial region segregation. (c) Rootogram that measures squared residual frequency for each tested model. *X*-axis is the count for gene *Mbp*. (d) Benchmarking five statistical models that capture mean and variance for each gene. Colors indicate different distributions. Note that the Zero inflated Poisson and GLM Poisson are overlapped with hurdle distribution. (e) Cell type clusters of 10× Visium Mouse Brain Anterior 1 using STew. (f) Top spatially informed gradients reflect the spatial domain variation. (g) Statistics of sCV1 associated genes based on Zero-inflated negative binomial modeling. Red color indicates statistical significance. (h) Gene expression pattern on the image for the selected genes that are significantly associated with sCV1.

To evaluate the scalability of STew, we first applied STew on mouse brain ST datasets generated from Slide-seqV2, almost single-cell resolution. For this mouse hippocampus data, it measured 41 786 locations generated from Slide-seqV2 (Stickels *et al.* 2020). We clustered on the spatially informed low-dimensions, top 20 sCVs, to identify different layers of mouse brain clusters (Fig. 3a, Supplementary Fig. S2). The identified biologically meaningful markers clearly reflect the regional dissociation of the tissue and are consistent with the known anatomic structures (Fig. 3b, Stickels *et al.* 2020). Next, we benchmarked five different statistical models with the potential to model overdispersed count data, aiming to pinpoint the right model for identifying significant genes associated with the discerned spatially informed axes or clusters.

Interestingly, both negative binomial and zero-inflated negative binomial models exhibited superior performance compared to the other models, indicated by Rootogram analysis to assess the goodness of model fit at the count level (Fig. 3c). Particularly, the Rootogram quantified squared residual frequency for gene *Mbp* (myelin basic proteins) counts, a major constituent of the myelin sheath of oligodendrocytes and Schwann cells in the nervous system. We observed that negative binomial and zero-inflated negative binomial models fitted the expression distribution very well (Fig. 3c) and best captured the mean and variance (Fig. 3d), but GLM Poisson, zero-inflated Poisson, and Hurdle underestimated the zero counts and overestimated the low counts (Supplementary Fig. S4). A subsequent analysis of the DLPFC dataset corroborated these findings that the gene expressions are optimally represented by either the negative binomial or the zero-inflated negative binomial distribution (Supplementary Fig. S5a).

Next, we applied STew on a mouse brain anterior dataset acquired by 10× Visium which contains 31 053 genes and 2696 locations, revealing spatially informed clusters (Fig. 3e) and distinct biologically meaningful gradients that capture different layers in the mouse brain (Fig. 3f, Supplementary Fig. S3). Taking sCV1 as an example, we presented the statistics generated from the zero-inflated negative binomial modeling. The genes with positive estimates, which are markers for the cells with positive loadings in sCV1, like gene *Cabp1* (Fig. 3g and h). Similarly, genes with negative estimates indicate markers for cells with negative loadings in sCV1. For example, gene *Adora2a* (Adenosine A2a Receptor) (Fig. 3h), an adenosine receptor group of G-protein-coupled receptors controlling synaptic plasticity, plays a critical role to modulate anxiety and sleep (Hohoff *et al.* 2020). Ultimately, we have incorporated this rigorous statistical model benchmarking within the STew framework, empowering users to automatically select the most appropriate model for analyzing ST features, which exhibit distributions usually depending on sequencing depth and technological resolution.

### 3.3 STew is scalable with thousands of spatial locations for single-cell resolution ST data

**Figure 4.**
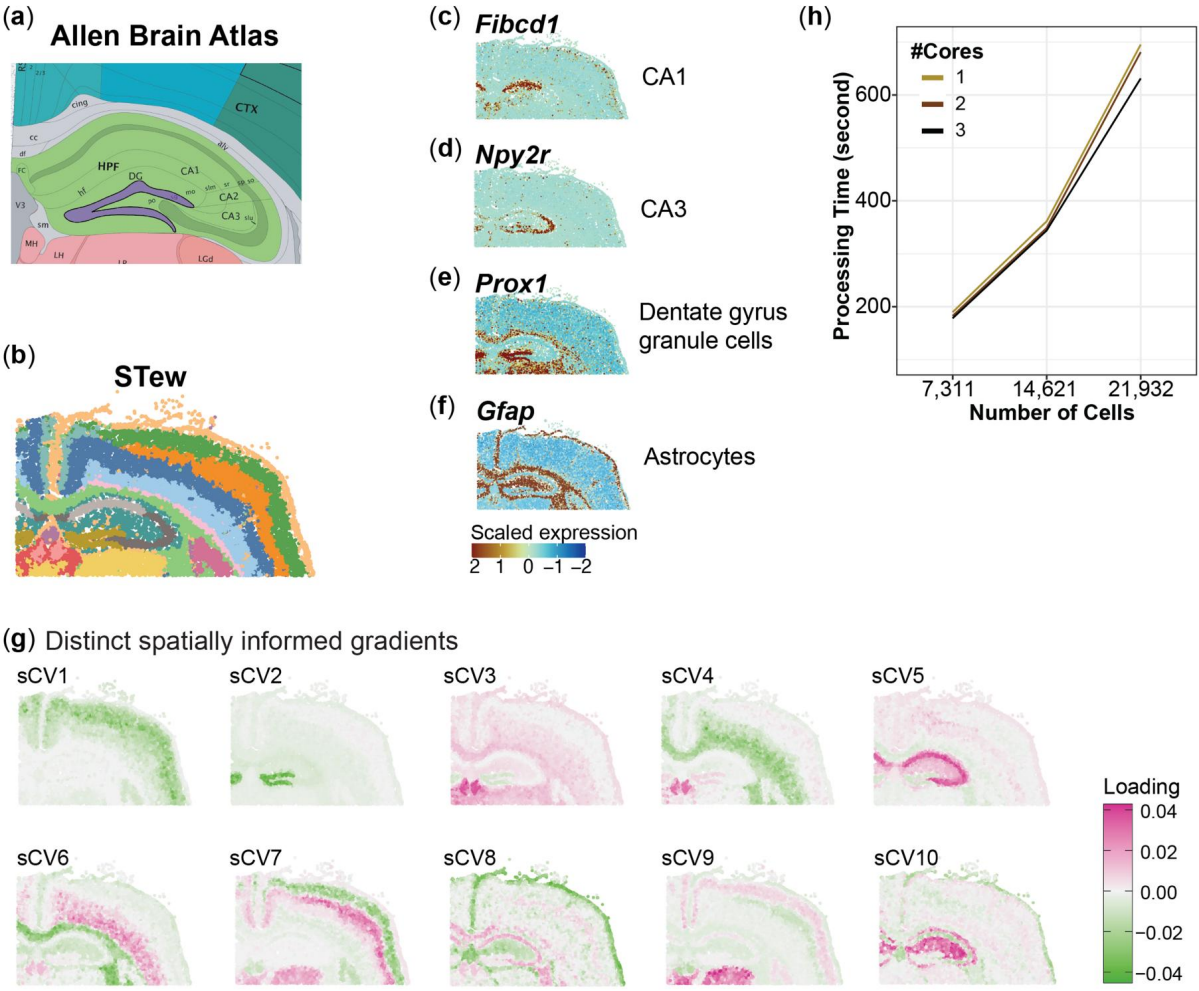
Applying STew on 10× Xenium Mouse Brain Coronal single-cell resolution spatial transcriptomic data. (a) Mouse Brain structure based on Allen Brain Atlas. (b) Identified clusters by STew. (c–f). Gene expression patterns for cell type cluster cornu ammonis 1, CA3, dentate gyrus granule cells, and astrocytes, respectively. (g) Identified sCVs (spatially informed canonical vectors). (h) Measuring scalability of STew by changing # cells based on 20%, 40%, and 60% random downsampling with different # computing cores.

To assess the capability of STew in analyzing single-cell resolution ST data, we applied it to the 10× Xenium Mouse Brain Coronal dataset, which quantifies 247 genes in 21 932 cells. We first show that STew is able to recapitulate well-described substructures of mouse coronal, as validated by annotations from the Allen Brain Atlas (Fig. 4a and b). For example, STew identifies cornu ammonis 1 subfield which highly expressed gene *Fibcd1*, and also CA3 of the hippocampus which highly expressed marker gene *Npy2r* (Fig. 4c and d). Similarly, *Prox1*, recognized for defining dentate gyrus granule cells (Lavado *et al.* 2010, Fig. 4e), along with *Gfap*, a well-established marker gene for astrocytes, highlighting its utility in studying astrocyte activation and roles in neuroinflammatory and neurodegenerative conditions (Escartin *et al.* 2021, Fig. 4f). Moreover, we further visualize learned sCVs within the mouse brain, showcasing the distinct variation captured from different spatial regions (Fig. 4g). A notable contribution of STew is the implementation of parallel computing for efficient data integration. By evaluating STew’s performance across various cell numbers via random downsampling, we observed an almost linear scalability with the number of spatial locations, enabling the completion of the full analytical pipeline for >21 000 cells in around 12 min (Fig. 4h). This computational efficiency is important for analyzing large single-cell resolved ST data, underscoring that STew is scalable to facilitate the identification of spatially distinct niches within complex tissue.

### 3.4 STew is sensitive to characterize the spatial organization of heterogeneous cell types in psoriatic skin

**Figure 5.**
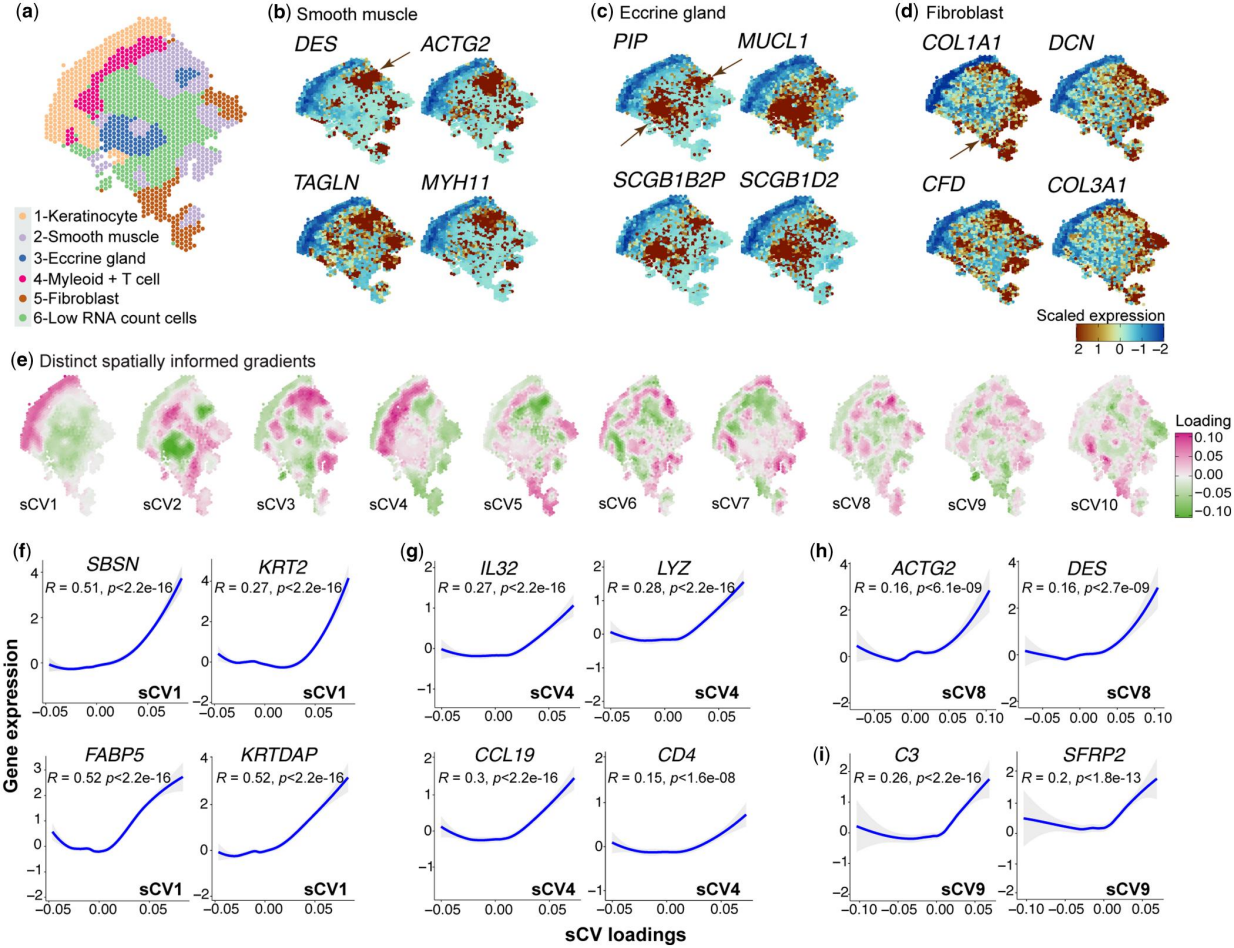
Analysis of psoriatic human skin spatial transcriptomics. (a) Identified cell type clusters. (b–d) Expressions of canonical cell type lineage marker genes for smooth muscle, eccrine gland, and fibroblast. (e) Top ten spatially informed gradients generated from STew. (f–i) Correlations between expressions of associated genes and specific sCVs. Pearson correlation and *P*-value with 95% CIs are given for each test.

Inflammatory diseases affect different human tissues which are usually more heterogeneous and complex than the well-structured human brain. To evaluate the sensitivity of STew, we applied it to analyze a recent ST dataset generated from psoriatic skin using 10× Genomics (Ma *et al.* 2023). For this psoriasis dataset that consists of 1352 cell spots, we first identified 525 spatially aggregated genes and applied STew to generate spatially informed sCVs. Using the top 20 sCVs, we identified six clusters and annotated them in alignment with canonical marker genes reported from the original study (Ma *et al.* 2023), including keratinocytes, smooth muscle cells, fibroblasts, eccrine glands, myeloid, and T cells, and a cluster characterized by a low RNA count (Fig. 5a). For instance, we are able to allocate smooth muscle cells, which aligned with corresponding marker gene expressions of *DES* (Pearson *r* = 0.18, *P *=* *3.4e-11), *ACTG2* (Pearson *r* = 0.19, *P *=* *1.2e-12), *TAGLN* (Pearson *r* = 0.33, *P *=* *2.2e-16), and *MYH11* (Pearson *r* = 0.14, *P *=* *9.5e-8) (Fig. 5b). In addition, we detected eccrine gland cells which were formed by two distant regions, but they consistently expressed the same marker genes, including *PIP*, *MUCL1*, *SCGB1B2P*, and *SCGB1D2* (Fig. 5c). We also identified fibroblasts that highly expressed canonical markers such as *COL1A1*, *DCN*, *CFD*, and *COL3A1* (Fig. 5d). Note that, fibroblasts, smooth muscle, and eccrine gland cells are very heterogeneous and mixed in the diseased skins, which are typically situated deeper within the dermis. This complexity renders them difficult to be distinguished using solely gene expression patterns, since the spatial location of these tissue-specific cell types can create unique microenvironments or niches. Our algorithm is able to delineate these cells into continuous and smooth spatial domains measured by a PAS value of 0.084, and further maintain the concordance with cell type lineage marker expressions (Fig. 5a–d). This analysis demonstrates the capability of STew in identifying the shared variations from both gene expression and anatomical spatial domains within the context of disease.

Next, we examined the identified low-dimensional components and identified genes significantly associated with these sCVs, which reflect biologically meaningful axes of spatial variation (Fig. 5e). For instance, genes associated with sCV1 (*n* = 504, FDR<0.05) capture the gradual changing of expressions for keratinocytes, including *SBSN*, *KRT2*, *FABP5*, and *KRTDAP* (Fig. 5f). Similarly, sCV4 loading is associated with gene expressions relevant to immune cells (*n* = 247, FDR<0.05), particularly myeloid cells (e.g. *LYZ*) and T cells (e.g. *CD4*) (Fig. 5g). Intriguingly, proinflammatory cytokines *CCL19* and *IL32,* detected by the statistical association from STew, are significantly associated with this axis as well, which further suggests an immune-derived spatial niche contributing to inflammatory disease pathogenesis (Pearson *r* = 0.3, *P *<* *2.2e-16) (Fig. 5g). In parallel, sCV8 explains the smooth muscle cell axis correlated with gene expressions like *ACTG2* and *DES* (*n* = 233, FDR<0.05) (Fig. 5h), and sCV9 reflects a potential fibroblast differentiation trajectory, as evidenced by the expression of *C3* and *SFRP2* (*n* = 422, FDR<0.05) (Fig. 5i). In particular, *SFRP2* is an important marker for the proinflammatory fibroblast subpopulation discovered in psoriatic skin, which reflects the transition from a fibrotic to an inflammatory stage (Ma *et al.* 2023). In all, our derived low-dimensional embeddings review spatial niches that are associated with disease pathology.

## 4 Discussion

Integrating spatial information into gene expression data is essential to understand the cellular activities in the tissue organization. In this study, we propose STew, a method for identifying the shared information between spatial cell neighborhood relationships and the covariance observed in cell–cell similarities from the gene expression space. Our hypothesis is grounded in the notion that adjacent cells tend to exhibit a greater resemblance in transcriptional identities (Nitzan *et al.* 2019, Ren *et al.* 2020, Wang *et al.* 2023). Through rigorous evaluation and benchmarking with other state-of-the-art algorithms on six single-cell ST datasets, STew demonstrated superior performance, particularly in terms of clustering accuracy, delineation of smooth and continuous spatial domains, and scalability. Furthermore, the representations derived from STew are effective in extracting biologically meaningful axes that reflect certain spatial niches, thereby facilitating the decryption of spatially informed cell neighborhood clusters. This progress marks a pivotal step towards a deeper understanding of the complex dynamics underlying cellular functions and tissue organization, opening new avenues for therapeutic intervention for disease.

Other ST integrative methods such as using deep learning-based models have been developed to decipher spatial domains by combining histological images with gene expression data (Pham *et al.* 2023, Zeng *et al.* 2023), but several potential drawbacks exist. One notable shortcoming is that this may lead to poor prediction when the image features drive construction of incorrect spot relationships, a problem exacerbated if the spot relations are not properly updated during the model training phase. Further, most of these models pass the crucial step of pretraining a specific big model on histological images for feature extraction. In addition, single-cell deep learning models frequently encounter issues with limited interpretability, and the embeddings they produce tend to be neither easily interpretable nor reproducible. In contrast, STew optimizes the covariance between gene expression patterns and spatial variations through a graph-embedded approach, which minimizes noise from individual data modalities and simultaneously learns representative features by enforcing sparsity constraints. Further, STew offers both scalability and interpretability, attributed to its linear characteristics, and the learned joint embeddings are reproducible given the optimization problem setting.

We note that the concept of using canonical correlation analysis has been used in genetics and microarray data integration (Parkhomenko *et al.* 2007, 2009), cross-dataset single-cell transcriptomics integration (Butler *et al.* 2018), and our recent CITE-seq multi-modal data integration strategy (Nathan *et al.* 2021, Zhang *et al.* 2023). STew effectively extends these ideas into a formal R package enriched with innovative functions specifically tailored to address ST data challenges, including (1) embedding the data points in the graph-based representations, (2) automatically estimating the sparsity penalties to infer the influence of spatial correlation, (3) scalable and reproducible to handle large ST datasets, and (4) integrating a robust statistical goodness of fit method into STew to elucidate the gene signatures that hold significant associations with joint embeddings or spatially informed clusters. Altogether, STew is a comprehensive analytical R tool for single-cell and multi-cellular resolution ST datasets.

Further, STew can be extended and improved in multiple aspects. One potential caveat of STew is its potential difficulty in identifying nonlinear patterns. To reveal nonlinear structures inherent in ST data (He *et al.* 2020, Biancalani *et al.* 2021, Zhao *et al.* 2023), one prospective avenue to explore is to extend this work by implementing nonlinear variations of canonical correlation analysis (Witten *et al.* 2009), such as incorporating kernels or autoencoder structures, thereby fostering the learning of extracted features through nonlinear processes. Other alternative strategies such as distance covariance analysis could help uncover both linear and nonlinear relationships (Cowley *et al*. 2017). Considering the instances where cells in proximate spatial locales may not consistently present analogous transcriptional patterns–due to the dynamic interplay between tissue structures and cell type heterogeneity–we could add the “private variables” (Wang *et al.* 2017) to help model unique variations from spatial region and gene expression space, supplementing the already captured common variation. Note that the field of multi-modal joint learning for ST data is still evolving, where balancing the measurement between correlation and discrimination poses a significant computational challenge. Therefore, methods that can maintain both sensitivity and specificity while capturing the covariance are needed to guide the integration process preventing overfitting and overcorrection. In addition, we expect that our statistical model framework in STew could be extended to account for cell type information, similar to the CTSV approach (Yu and Luo 2022). As the volume of ST datasets continues to grow, we anticipate that STew will facilitate the discovery of new cellular organization to help researchers identify new disease mechanisms.

## Supplementary Material

vbae064_Supplementary_Data

## Code availability

The code and tutorials for STew package are available at https://github.com/fanzhanglab/STew.

## References

[vbae064-B1] Anselin L. Local indicators of spatial association—LISA. Geogr Anal 2010;27:93–115.

[vbae064-B2] Biancalani T , Scalia G, Buffoni L et al Deep learning and alignment of spatially resolved single-cell transcriptomes with tangram. Nat Methods 2021;18:1352–62. 34711971 10.1038/s41592-021-01264-7 PMC8566243

[vbae064-B3] Butler A , Hoffman P, Smibert P et al Integrating single-cell transcriptomic data across different conditions, technologies, and species. Nat Biotechnol 2018;36:411–20. 29608179 10.1038/nbt.4096 PMC6700744

[vbae064-B4] Chen K , Boettiger AN, Moffitt JR et al RNA imaging. Spatially resolved, highly multiplexed RNA profiling in single cells. Science 2015;348:aaa6090. 25858977 10.1126/science.aaa6090 PMC4662681

[vbae064-B5] Cowley B , Semedo J, Zandvakili A et al Distance covariance analysis. In: *Proceedings of the 20th International Conference on Artificial Intelligence and Statistics, Fort Lauderdale, FL, Volume 54*. Proceedings of Machine Learning Research (PMLR), 2017, 242–51.

[vbae064-B6] Dries R , Zhu Q, Dong R et al Giotto: a toolbox for integrative analysis and visualization of spatial expression data. Genome Biol 2021;22:78. 33685491 10.1186/s13059-021-02286-2 PMC7938609

[vbae064-B7] Ebrahimi M , Yamamoto Y, Sharifi K et al Astrocyte-expressed FABP7 regulates dendritic morphology and excitatory synaptic function of cortical neurons. Glia 2016;64:48–62. 26296243 10.1002/glia.22902

[vbae064-B8] Escartin C , Galea E, Lakatos A et al Reactive astrocyte nomenclature, definitions, and future directions. Nat Neurosci 2021;24:312–25. 33589835 10.1038/s41593-020-00783-4 PMC8007081

[vbae064-B9] Hao Y , Stuart T, Kowalski MH et al Dictionary learning for integrative, multimodal and scalable single-cell analysis. Nat Biotechnol 2023;42:293–304. 37231261 10.1038/s41587-023-01767-y PMC10928517

[vbae064-B10] He B , Bergenstråhle L, Stenbeck L et al Integrating spatial gene expression and breast tumour morphology via deep learning. Nat Biomed Eng 2020;4:827–34. 32572199 10.1038/s41551-020-0578-x

[vbae064-B11] Hohoff C , Kroll T, Zhao B et al ADORA2A variation and adenosine A1 receptor availability in the human brain with a focus on anxiety-related brain regions: modulation by ADORA1 variation. Transl Psychiatry 2020;10:406–11. 33235193 10.1038/s41398-020-01085-w PMC7686488

[vbae064-B12] Janesick A , Shelansky R, Gottscho A et al High resolution mapping of the tumor microenvironment using integrated single-cell, spatial and in situ analysis. Nat Commun 2023;14:1. 38114474 10.1038/s41467-023-43458-x PMC10730913

[vbae064-B13] Ke R , Mignardi M, Pacureanu A et al In situ sequencing for RNA analysis in preserved tissue and cells. Nat Methods 2013;10:857–60. 23852452 10.1038/nmeth.2563

[vbae064-B14] Kleiber C , Zeileis A. Visualizing count data regressions using rootograms. Am Stat 2016;70:296–303.

[vbae064-B15] Larsson L , Frisén J, Lundeberg J. Spatially resolved transcriptomics adds a new dimension to genomics. Nat Methods 2021;18:15–8. 33408402 10.1038/s41592-020-01038-7

[vbae064-B16] Lavado A , Lagutin OV, Chow LM et al Prox1 is required for granule cell maturation and intermediate progenitor maintenance during brain neurogenesis. PLoS Biol 2010;8:e1000460. 10.1371/journal.pbio.1000460 20808958 PMC2923090

[vbae064-B17] Li Y , Yang M, Zhang Z. A survey of multi-view representation learning. IEEE Trans Knowl Data Eng 2019;31:1863–83.

[vbae064-B18] Ma F , Plazyo O, Billi AC et al Single cell and spatial sequencing define processes by which keratinocytes and fibroblasts amplify inflammatory responses in psoriasis. Nat Commun 2023;14:1–19. 37308489 10.1038/s41467-023-39020-4 PMC10261041

[vbae064-B19] Maynard KR , Collado-Torres L, Weber LM et al Transcriptome-scale spatial gene expression in the human dorsolateral prefrontal cortex. Nat Neurosci 2021;24:425–36. 33558695 10.1038/s41593-020-00787-0 PMC8095368

[vbae064-B20] McFadden D. Conditional logit analysis of qualitative choice behavior. November 1972. https://escholarship.org/uc/item/61s3q2xr (1 March 2024, date last accessed).

[vbae064-B21] Miller BF , Bambah-Mukku D, Dulac C et al Characterizing spatial gene expression heterogeneity in spatially resolved single-cell transcriptomics data with nonuniform cellular densities. Genome Res 2021;31:1843–55. 10.1101/gr.271288.120 34035045 PMC8494224

[vbae064-B22] Moffitt JR , Hao J, Bambah-Mukku D et al High-performance multiplexed fluorescence in situ hybridization in culture and tissue with matrix imprinting and clearing. Proc Natl Acad Sci USA 2016;113:14456–61. 27911841 10.1073/pnas.1617699113 PMC5167177

[vbae064-B23] Moses L , Pachter L. Museum of spatial transcriptomics. Nat Methods 2022;19:534–46. 35273392 10.1038/s41592-022-01409-2

[vbae064-B24] Nathan A , Beynor JI, Baglaenko Y et al Multimodally profiling memory T cells from a tuberculosis cohort identifies cell state associations with demographics, environment and disease. Nat Immunol 2021;22:781–93. 34031617 10.1038/s41590-021-00933-1 PMC8162307

[vbae064-B25] Nitzan M , Karaiskos N, Friedman N et al Gene expression cartography. Nature 2019;576:132–7. 31748748 10.1038/s41586-019-1773-3

[vbae064-B26] Papalexi E , Satija R. Single-cell RNA sequencing to explore immune cell heterogeneity. Nat Rev Immunol 2018;18:35–45. 28787399 10.1038/nri.2017.76

[vbae064-B27] Pardo B , Spangler A, Weber LM et al spatialLIBD: an R/bioconductor package to visualize spatially-resolved transcriptomics data. BMC Genomics 2022;23:434. 35689177 10.1186/s12864-022-08601-w PMC9188087

[vbae064-B28] Parkhomenko E , Tritchler D, Beyene J. Genome-wide sparse canonical correlation of gene expression with genotypes. BMC Proc 2007;1(Suppl 1):S119. 18466460 10.1186/1753-6561-1-s1-s119 PMC2367499

[vbae064-B29] Parkhomenko E , Tritchler D, Beyene J. Sparse canonical correlation analysis with application to genomic data integration. Stat Appl Genet Mol Biol 2009;8:Article 1. 19222376 10.2202/1544-6115.1406

[vbae064-B30] Pham D , Tan X, Balderson B et al Robust mapping of spatiotemporal trajectories and cell-cell interactions in healthy and diseased tissues. Nat Commun 2023;14:7739. 38007580 10.1038/s41467-023-43120-6 PMC10676408

[vbae064-B32] Ren H , Walker BL, Cang Z et al Identifying multicellular spatiotemporal organization of cells with SpaceFlow. Nat Commun 2022;13:1–14. 35835774 10.1038/s41467-022-31739-w PMC9283532

[vbae064-B33] Ren X , Zhong G, Zhang Q et al Reconstruction of cell spatial organization from Single-Cell RNA sequencing data based on ligand-receptor mediated self-assembly. Cell Res 2020;30:763–78. 32541867 10.1038/s41422-020-0353-2 PMC7608415

[vbae064-B34] Rodriques SG , Stickels RR, Goeva A et al Slide-Seq: a scalable technology for measuring genome-wide expression at high spatial resolution. Science 2019;363:1463–7. 30923225 10.1126/science.aaw1219 PMC6927209

[vbae064-B35] Shalek AK , Benson M. Single-cell analyses to tailor treatments. Sci Transl Med 2017;9:eaan4730. 10.1126/scitranslmed.aan4730 28931656 PMC5645080

[vbae064-B36] Shang L , Zhou X. Spatially aware dimension reduction for spatial transcriptomics. Nat Commun 2022;13:7203. 36418351 10.1038/s41467-022-34879-1 PMC9684472

[vbae064-B37] Ståhl PL , Salmén F, Vickovic S et al Visualization and analysis of gene expression in tissue sections by spatial transcriptomics. Science 2016;353:78–82. 27365449 10.1126/science.aaf2403

[vbae064-B38] Stickels RR , Murray E, Kumar P et al Highly sensitive spatial transcriptomics at near-Cellular resolution with slide-seqV2. Nat Biotechnol 2020;39:313–9. 33288904 10.1038/s41587-020-0739-1 PMC8606189

[vbae064-B39] Stubbington MJT , Rozenblatt-Rosen O, Regev A et al Single-cell transcriptomics to explore the immune system in health and disease. Science 2017;358:58–63. 28983043 10.1126/science.aan6828 PMC5654495

[vbae064-B40] Vandereyken K , Sifrim A, Thienpont B et al Methods and applications for single-cell and spatial multi-omics. Nat Rev Genet 2023;24:494–515. 36864178 10.1038/s41576-023-00580-2 PMC9979144

[vbae064-B41] Wang R , Peng G, Tam PPL et al Integration of computational analysis and spatial transcriptomics in single-cell studies. Genomics Proteomics Bioinformatics 2023;21:13–23. 35901961 10.1016/j.gpb.2022.06.006 PMC10372908

[vbae064-B42] Wang W , Yan XLee H, Livescu K. Deep variational canonical correlation analysis. arXiv:1610.03454, 2017. 10.48550/arXiv.1610.03454.

[vbae064-B43] Williams CG , Lee H, Asatsuma T et al An introduction to spatial transcriptomics for biomedical research. Genome Med 2022;14:68. 35761361 10.1186/s13073-022-01075-1 PMC9238181

[vbae064-B44] Witten DM , Tibshirani R, Hastie T. A penalized matrix decomposition, with applications to sparse principal components and canonical correlation analysis. Biostatistics 2009;10:515–34. 19377034 10.1093/biostatistics/kxp008 PMC2697346

[vbae064-B45] Yu J , Luo X. Identification of cell-type-specific spatially variable genes accounting for excess zeros. Bioinformatics 2022;38:4135–44. 35792822 10.1093/bioinformatics/btac457 PMC9438960

[vbae064-B46] Zeng Y , Yin R, Luo M et al Identifying spatial domain by adapting transcriptomics with histology through contrastive learning. Brief Bioinform 2023;24:bbad048. 36781228 10.1093/bib/bbad048

[vbae064-B47] Zhang F , Jonsson AH, Nathan A et al; Accelerating Medicines Partnership: RA/SLE Network. Deconstruction of rheumatoid arthritis synovium defines inflammatory subtypes. Nature 2023;623:616–24. 37938773 10.1038/s41586-023-06708-y PMC10651487

[vbae064-B48] Zhao Y , Wang K, Hu G. DIST: spatial transcriptomics enhancement using deep learning. Brief Bioinform 2023;24:bbad013. 10.1093/bib/bbad013 36653906

