# Supplementary material for: Uncover spatially informed variations for single-cell spatial transcriptomics with STew
Source: Bioinform Adv. 2024 May 29;4(1):vbae064. doi: 10.1093/bioadv/vbae064 (PMC11142628; doi:10.1093/bioadv/vbae064)
Supplement: vbae064_Supplementary_Data [file vbae064_supplementary_data.docx]

**Supplementary Information**

**Title: Uncover Spatially Informed Variations for Single-Cell Spatial Transcriptomics with STew**

**Author**: Nanxi Guo^1,2^, Juan Vargas^2,3^, Samantha Reynoso^2,4^, Douglas Fritz^2,5^, Revanth Krishna^2,6^, Chuangqi Wang^7^, Fan Zhang^2,6^


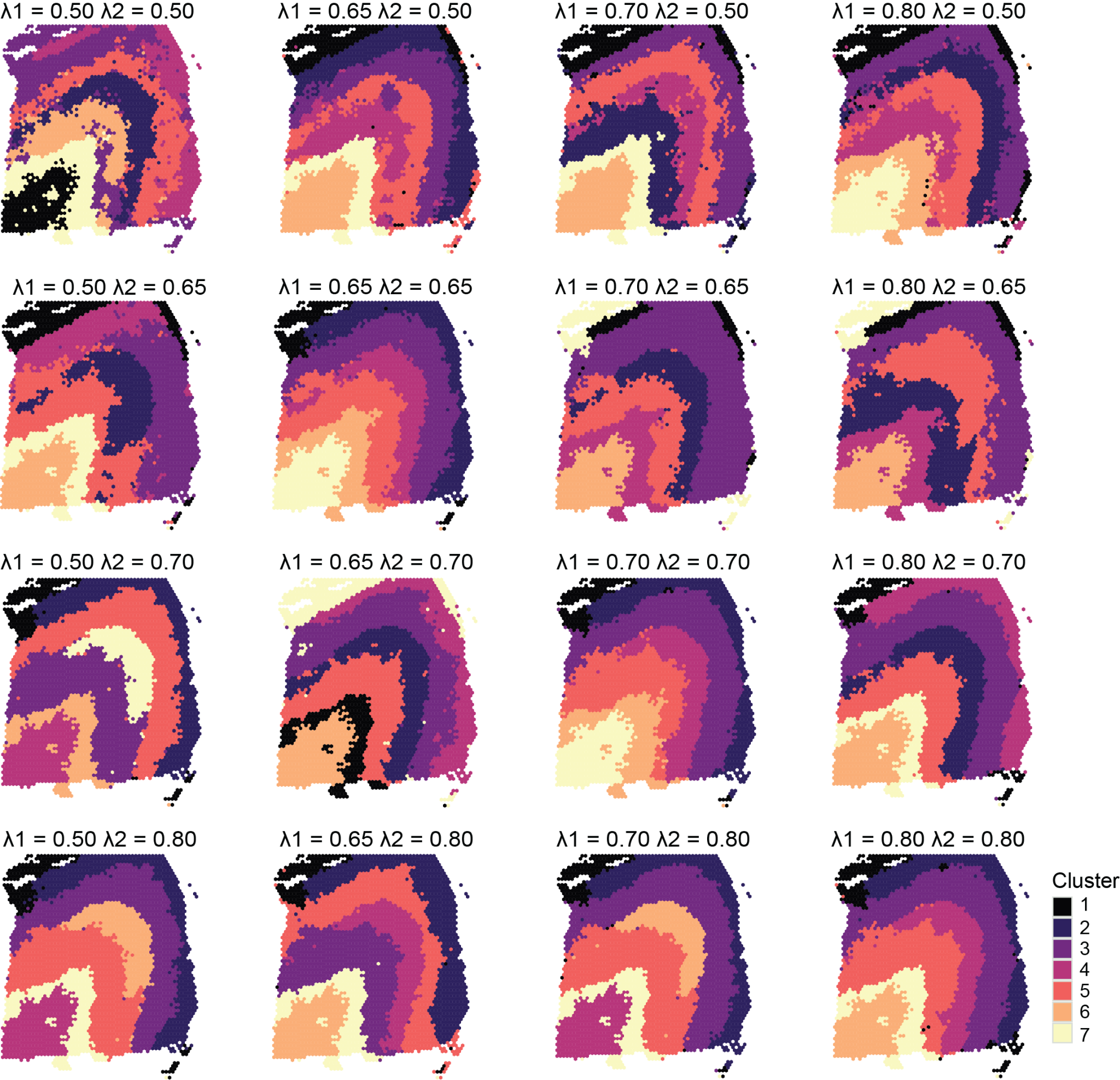


**Supplementary Figure 1.** **Sensitivity analyses of running STew on the DLPFC data.** Identified cell type clusters from STew are shown based on different sparsity constraints. $\lambda_{1}$ denotes the penalty of gene expression-driven adjacency matrix. $\lambda_{2}$ denotes the penalty of spatial data-driven cell neighborhood graph.


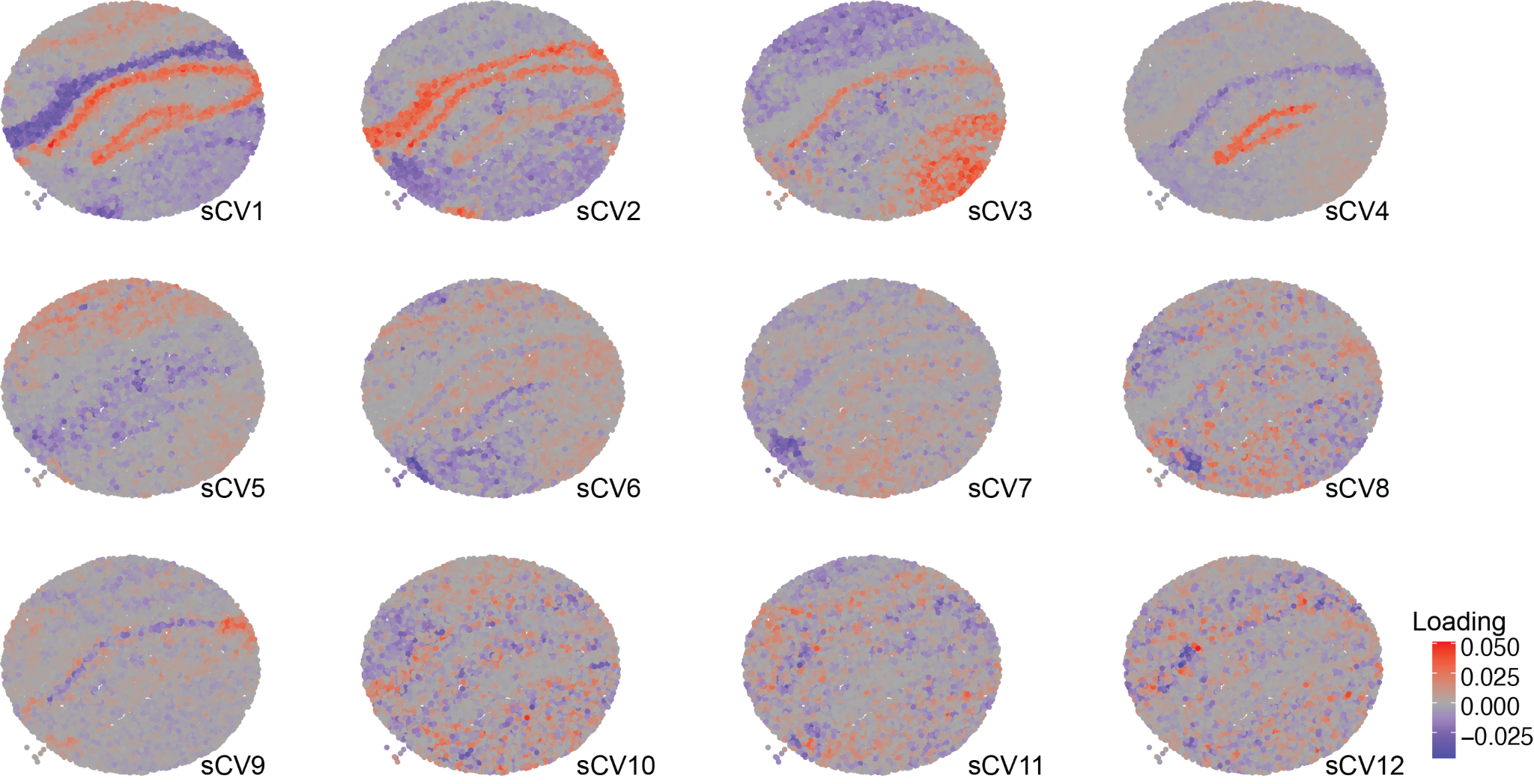


**Supplementary Figure 2.** **Spatially informed gradients from STew on mouse hippocampus data from Slide-seqV2.** Different spatially informed sCVs are visualized.


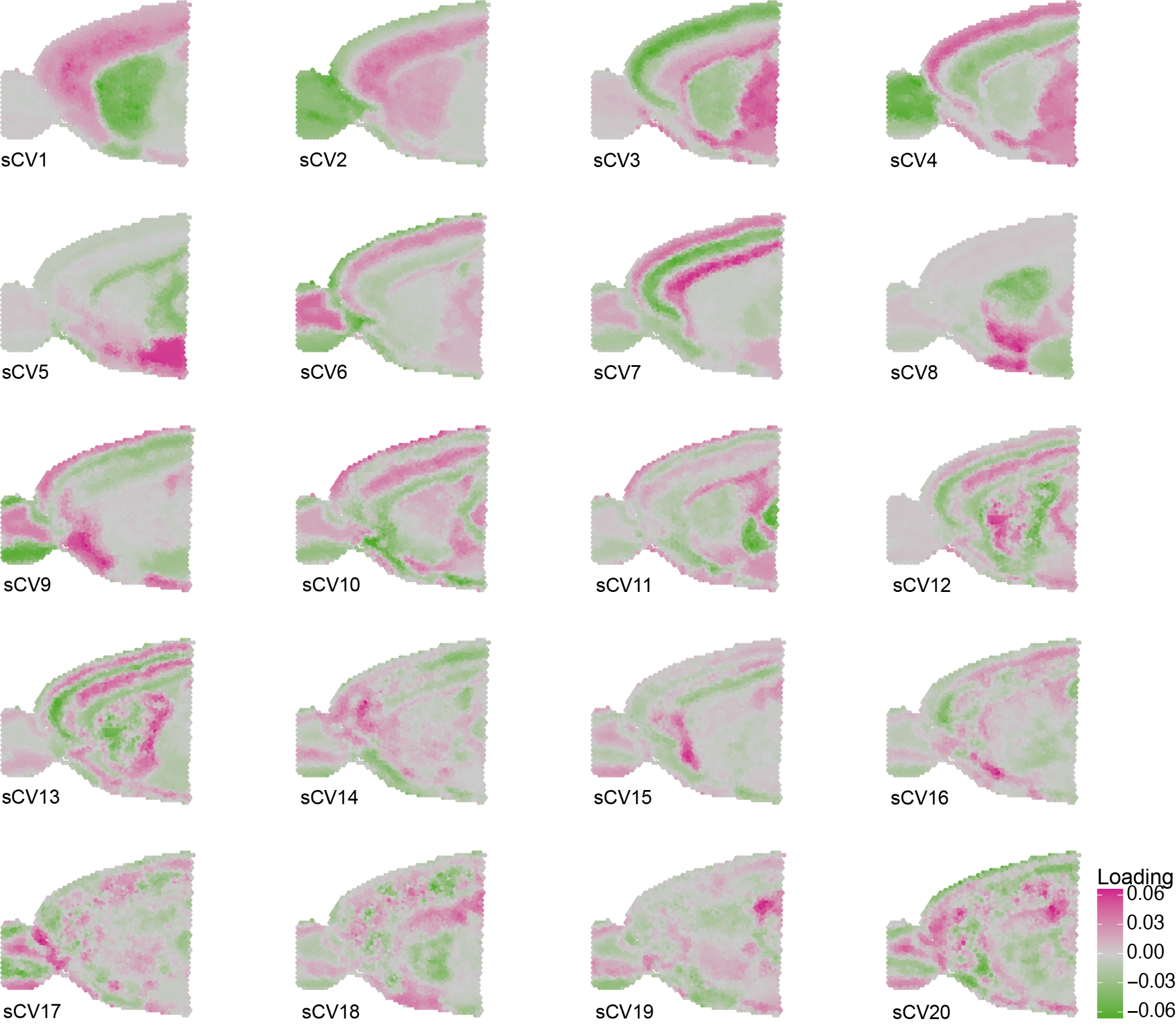


**Supplementary Figure 3.** **Spatially informed gradients from STew on mouse brain anterior data from 10x Visium**. Different spatially informed sCVs are visualized.


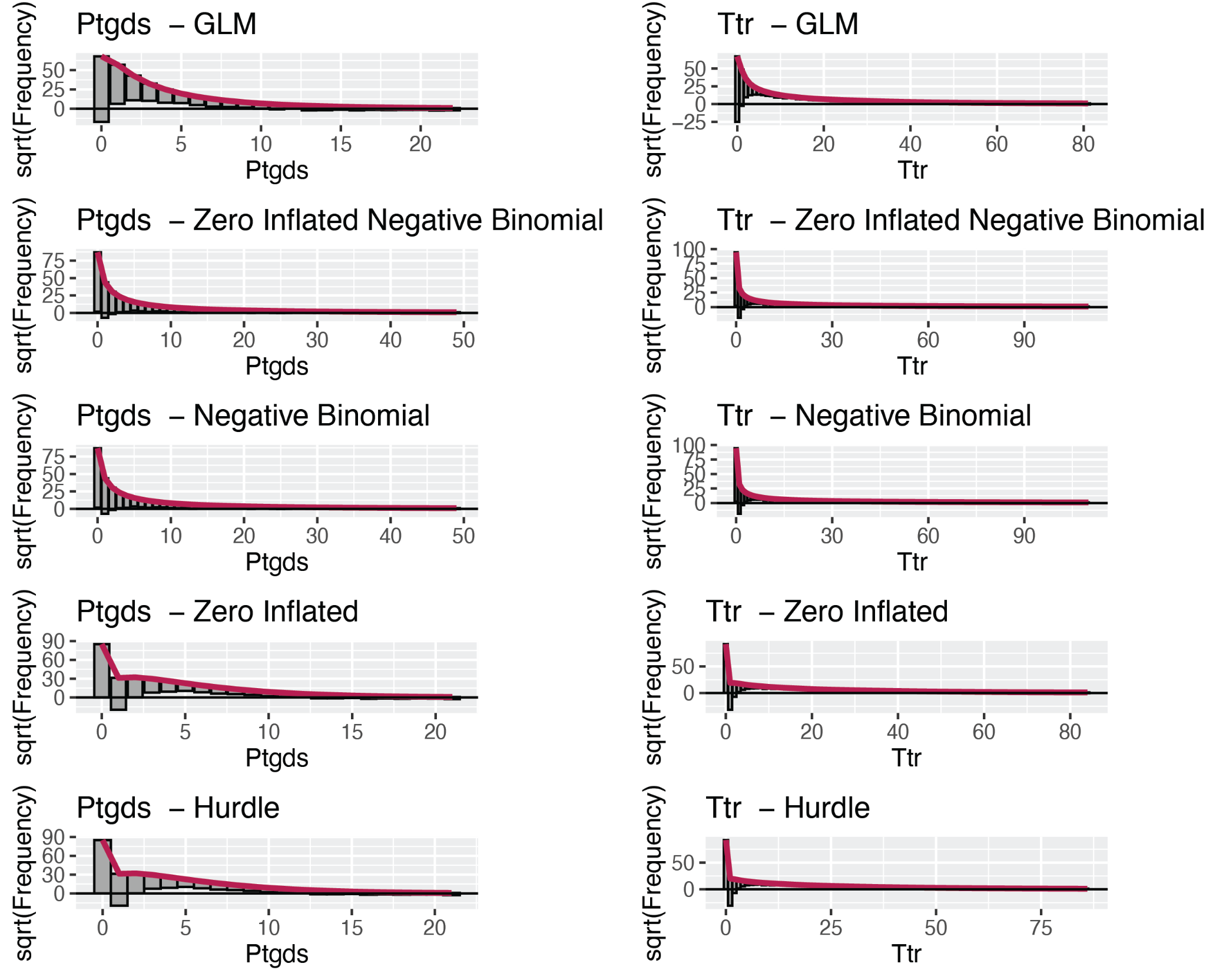


**Supplementary Figure 4.** **Evaluation of statistical model performance**. Rootogram plots on *ptgds* and *Ttr* that measure squared residual frequency on the DLPFC dataset (Sample ID 151673) for each tested model.


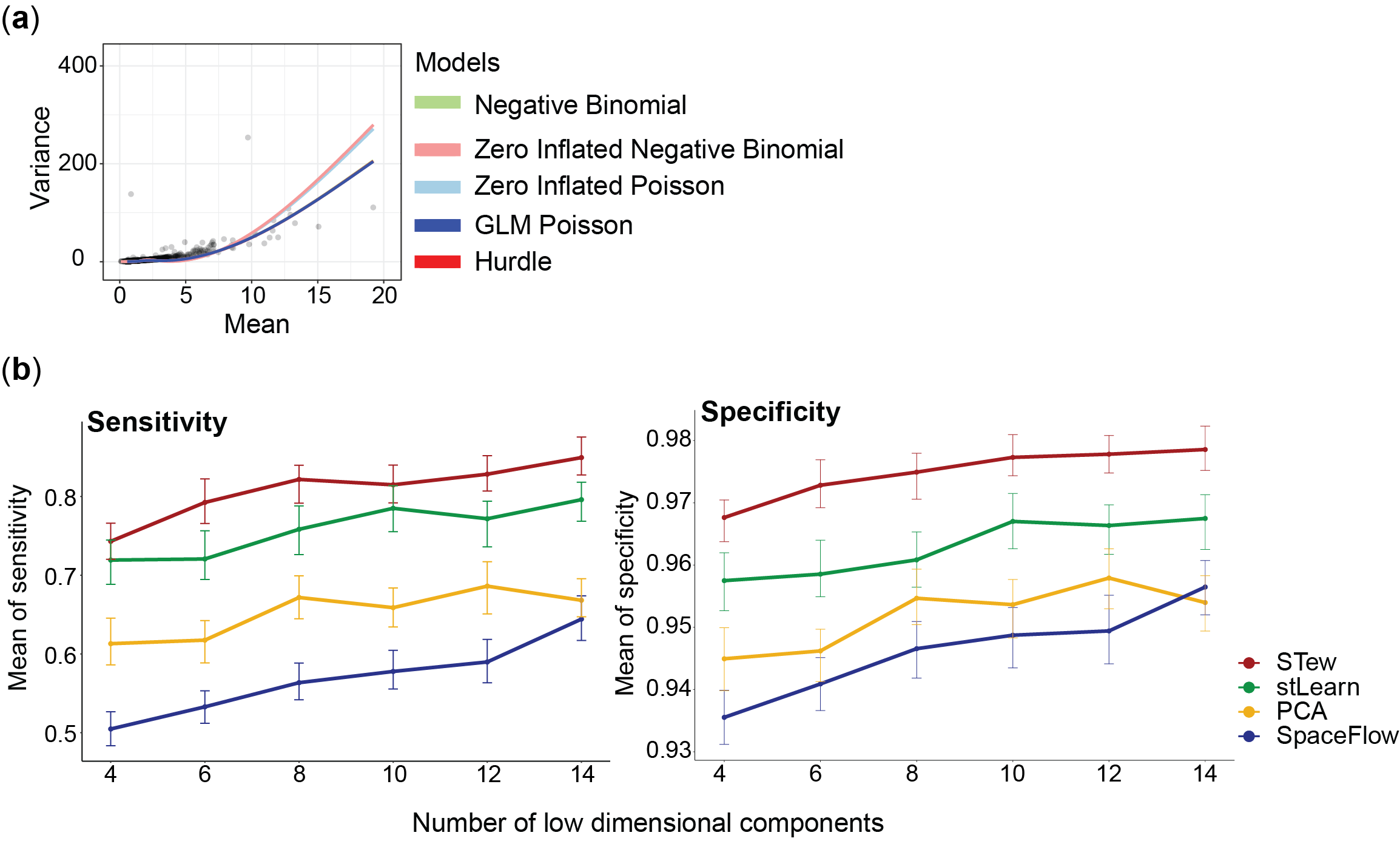


**Supplementary Figure 5. Performance evaluation on the DLPFC benchmarking analysis.** (**a**) Benchmarking five statistical models that capture mean and variance for each of the tested 1,500 genes. Colors indicate different distributions. Note that the Negative Binomial and GLM Poisson are overlapped with hurdle distribution. (**b**) Evaluation of the low dimensional components regarding their capability to predict the ground truth of tissue regions measured by sensitivity and specificity (see Methods). 95% confidence Intervals are calculated based on bootstrapping.


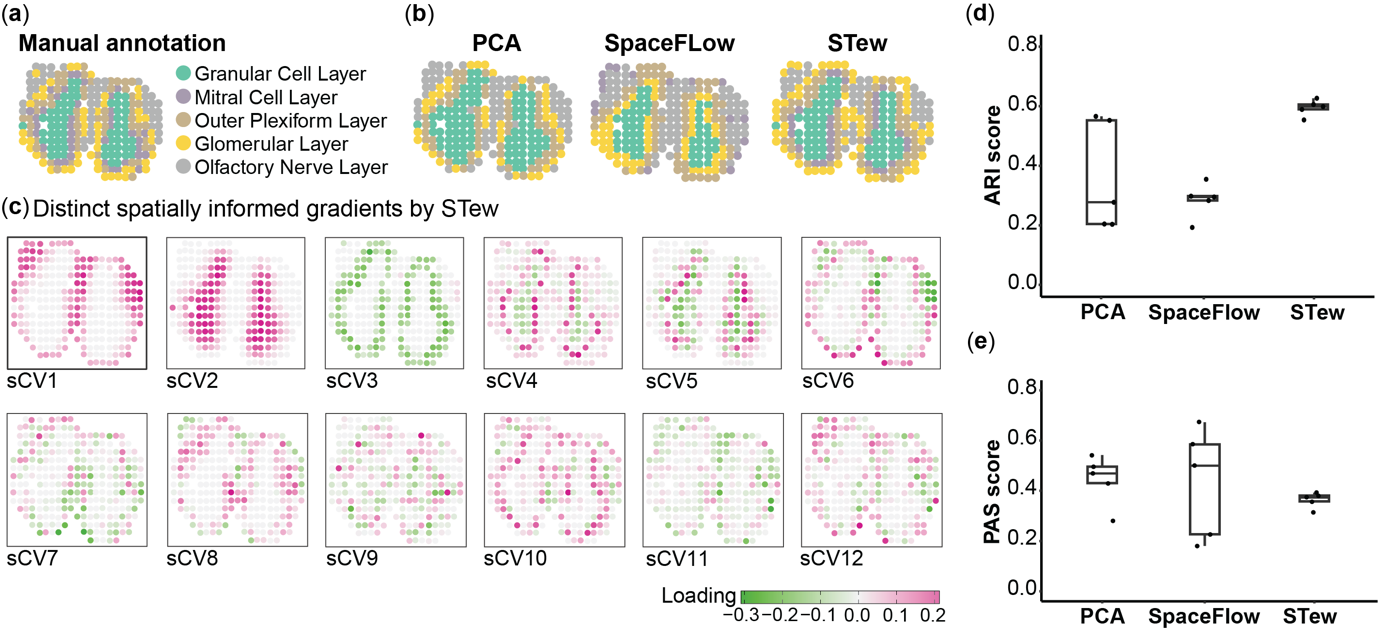


**Supplementary Figure 6. Applying STew on the Mouse Main Olfactory Bulb (MOB) spatial transcriptomic dataset.** (**a**) Manual annotated cell type clusters for MOB. (**b**) Identified clusters by PCA, SpaceFlow, and STew. (**c**) Distinct spatially informed gradients identified by STew. (**d**) Clustering accuracy by comparwith with manual labels measured by ARI, with different numbers of low dimensions in each of the algorithms, in particular top 12,14, 16, 18 and 20. In the boxplot, the center line, box limits and whiskers denote the median, upper, and lower quartiles, and 1.5 interquartile range, respectively. (**e**) Percentage of abnormal spots (PAS), the proportion of spots with a cluster label that is different from at least six of its neighboring ten spots.


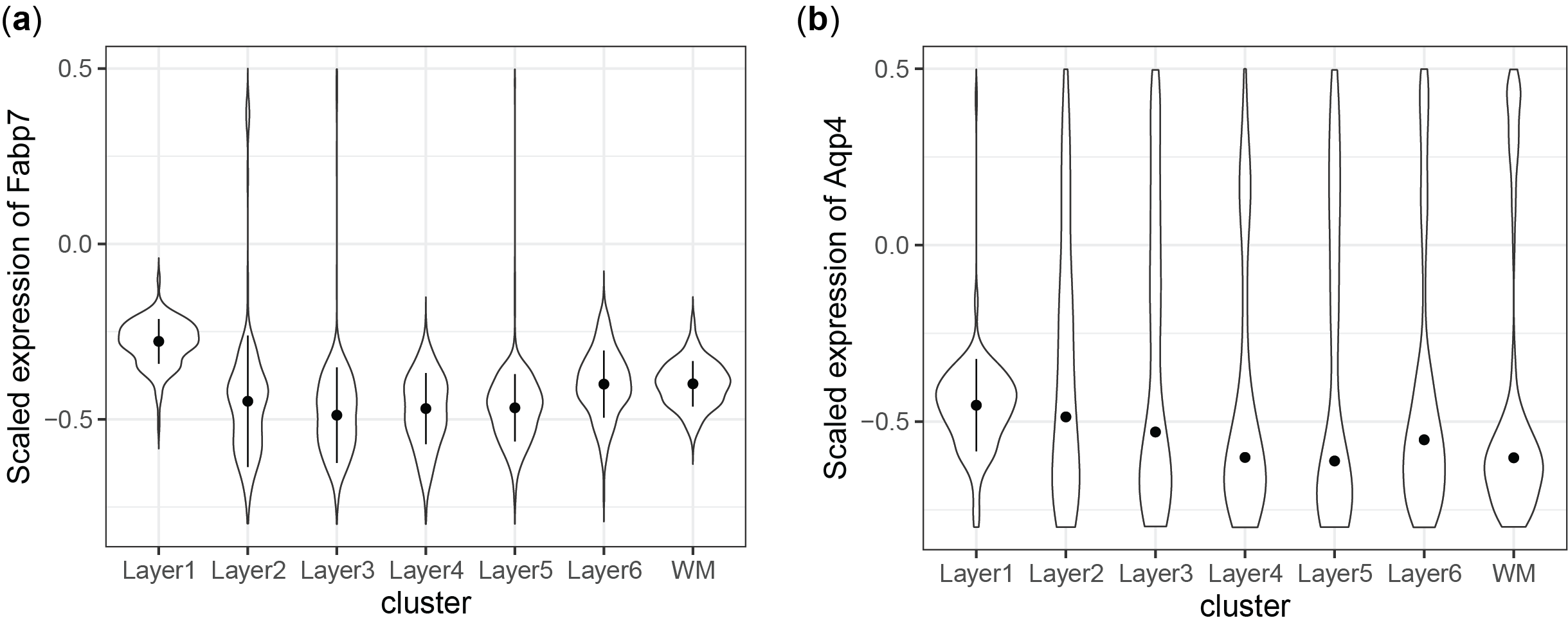


**Supplementary Figure 7**. **Boxplots of normalized gene expression for layer1-specific genes** for **(a**) *Fabp7* and (**b**) *AQP4*.
